# Profil infectieux et mortalité des enfants âgés de 0 à 5 ans admis pour malnutrition aiguë sévère: étude de cohorte rétrospective au Centre Nutritionnel et Thérapeutique de Bukavu, République Démocratique du Congo

**DOI:** 10.11604/pamj.2016.23.139.8370

**Published:** 2016-03-28

**Authors:** Richard Mbusa Kambale, Joe Bwija Kasengi, John Mutendela Kivukuto, Liévin Murhula Cubaka, Bruno Masumbuko Mungo, Ghislain Bisimwa Balaluka

**Affiliations:** 1 Université Catholique de Bukavu (UCB), Bukavu, République Démocratique du Congo; 2 Hôpital Provincial Général de Référence de Bukavu (HPGRB), Bukavu, République Démocratique du Congo; 3 ONG-MDA, Coordination Europe, Savigny-sur-Orge, France

**Keywords:** Malnutrition, enfants, infections, mortalité infantile, Malnutrition, children, infections, infant mortality

## Abstract

**Introduction:**

La malnutrition constitue une toile de fond sur laquelle se greffent plusieurs infections. L'objectif de ce travail est de déterminer les infections les plus létales, la durée médiane de séjour et le gain pondéral médian journalier des enfants malnutris.

**Méthodes:**

Étude de cohorte rétrospective des enfants malnutris de 0 à 59 mois hospitalisés au Centre Nutritionnel et Thérapeutique de Bukavu du 1^er^ janvier 2011 au 31 décembre 2013. L’évaluation du risque de survenue de décès par complication infectieuse a été faite par la mesure du risque relatif. Nous avons utilisé le test de Mann-Whitney pour comparer les médianes. Les Odd ratio ajustés par régression logistique et leurs intervalles de confiance à 95% du risque de mortalité ont été donnés pour chaque cause infectieuse.

**Résultats:**

Au total, 574 enfants avaient été inclus. Cinq cent vingt et un (90.8%) enfants étaient sortis guéris, 10 (1.7%) avaient abandonné le traitement et 43 (7.5%) étaient décédés. La durée médiane de séjour était de 19 (13-26) jours et le gain pondéral médian journalier était de 7 (3-13) g/kg/j. Il existait une association statistiquement significative entre la mortalité et le sepsis / choc septique (p = 0,0004), la méningite (p = 0,00001), et l'infection à VIH (p = 0,02).

**Conclusion:**

Une meilleure prise en charge de la malnutrition aigüe dans notre région devrait se baser sur la mise en place des unités spécialisées et bien équipées pour la prise en charge de la malnutrition associées aux infections sévères.

## Abstract (en)

**Introduction:**

Malnutrition is a backdrop on which several infections are grafted. The aim of this study is to determine the most lethal infections, the median length of stay and the median daily weight gain of malnourished children.

**Method:**

A retrospective cohort study of malnutrition in children aged 0-59 months hospitalized in the Therapeutic Nutritional Center in Bukavu from 1 January 2011 to 31 December 2013. The evaluation of the risk of deaths related to infectious complication was made measuring the relative risk. Mann-Whitney test was used for comparing the medians. Adjusted odd ratios using logistic regression and 95% confidence interval for the risk of mortality were given for each infectious cause.

**Results:**

A total of 574 children were included in the study. Five hundred twenty-one (90.8%) children were cured, 10 (1.7%) had discontinued treatment and 43 (7.5%) had died. The median length of stay was 19 (13-26) days and the median daily weight gain was 7 (3-13) g/kg/j. There was a statistically significant association between mortality and sepsis/septic shock (p = 0.0004), meningitis (p = 0.00001) and HIV infection (p = 0.02).

**Conclusion:**

A better management of acute malnutrition in our region should be based on the establishment of specialized and well equipped units for the treatment of malnutrition associated with severe infections.

## Introduction

La malnutrition reste un véritable problème de santé publique dans la plupart des pays en développement. En 2014, le taux de la malnutrition aiguë sévère (MAS) était estimé à 9.8% dans la Région Africaine de l'OMS [1]. En République Démocratique du Congo (RDC), bien que la prévalence de la malnutrition aiguë ait globalement diminué (16% en 2001, 11% en 2010 et 7.9% en 2014), elle dépasse toujours le seuil critique de 10% dans plusieurs provinces [2]. Les comorbidités telles que les infirmités motrices cérébrales, les cardiopathies congénitales et le syndrome de Down constituent des facteurs pronostiques de mortalité. Il est connu que la MAS est associée à une gravité accrue des maladies infectieuses courantes et que le décès des enfants atteints de MAS survient presque toujours à la suite d'une infection [3]. Dans certains pays en développement, ces décès affectent plus de 70% d'enfants de moins 5 ans [4]. Les infections les plus létales chez les enfants malnutris restent inconnues dans notre milieu. L'objectif principal de ce travail est de les identifier en vue de permettre au clinicien de mieux les prévenir. Secondairement, ce travail vise à apprécier la durée médiane de séjour et le gain pondéral médian journalier des enfants malnutris infectés.

## Méthodes

Il s'agissait d'une étude de cohorte rétrospective. Elle a couvert une période de 36 mois, allant de janvier 2011 à décembre 2013. Elle a été réalisée au Centre Nutritionnel et Thérapeutique (CNT) de l'Hôpital Provincial Général de Référence de Bukavu (HPGRB). Ce centre s'occupe de la prise en charge des enfants souffrant de MAS associée aux complications médicales. Etant situé dans un hôpital de niveau tertiaire sur la pyramide de la santé, le CNT reçoit, non seulement les enfants malnutris provenant directement de leurs domiciles, mais aussi ceux transférés par tous les autres hôpitaux de Bukavu. Étaient inclus dans l’étude tous les enfants âgés de 0 et 5 ans admis au CNT pour une MAS et disposant d'un dossier médical complet. Notre échantillon a été subdivisé en 2 classes: la classe des enfants de moins de 2 ans, supposée comme la plus à risque de MAS et de décès à cause des contraintes liées à l'alimentation (allaitement maternel exclusif jusqu’à 6 mois, contraintes liées à la qualité de la diversification alimentaire et de la poursuite de l'allaitement jusqu’à 2 ans), et celle des enfants entre 2 ans et 5 ans supposée être la classe à faible risque. Les données sociodémographiques (âge, sexe), anthropométriques (poids, taille, périmètre brachial), cliniques (diagnostic infectieux) et biologiques (sérologie VIH, goutte épaisse, examen cytobactériologique du LCR ou des urines, radiographie pulmonaire) ont été recueillies chez ces enfants à leur admission, durant leur séjour et à leur sortie au CNT. La MAS était définie selon les critères de l'OMS 2006: un indice poids par rapport à la taille < - 3 z-scores et / ou un périmètre brachial < 115 mm et / ou la présence d’œdèmes bilatéraux nutritionnels [5]. La gastro-entérite était simplement définie par la présence des signes fonctionnels digestifs à type de diarrhées et de vomissements; le paludisme était défini par une goutte épaisse positive; les infections des voies respiratoires inférieures étaient une entité regroupant les bronchites, les bronchiolites, les pneumonies et les bronchopneumonies selon la définition clinique ou radiologique retrouvée dans le dossier médical; les infections des voies respiratoires supérieures regroupaient les rhinopharyngites, les angines et les otites moyennes aiguës telles que notées cliniquement. Du fait de la difficulté de mise en évidence du bacille de Koch chez l'enfant dans nos conditions de travail, la tuberculose était définie par les symptômes et signes évocateurs associés à des anomalies radiologiques évocatrices [6]. Le sepsis était défini par l'association d'au moins deux critères du syndrome de réponse inflammatoire systémique (fièvre ou hypothermie, polypnée ou bradypnée, tachycardie, hyperleucocytose ou leucopénie) avec un foyer infectieux bien identifié [7]. Le choc septique était défini par les éléments de sepsis associés à une ou plusieurs dysfonctions d'organes (circulatoire, respiratoire, rénale, hépatique, les troubles des fonctions supérieures et de la coagulation) et à une hypotension [7]. En raison de l'indisponibilité de tests de diagnostic virologique précoce dans notre milieu, l'infection à VIH était retenue soit sur base d'une sérologie VIH positive après l’âge de 18 mois, soit sur base des signes cliniques évocateurs de l'infection à VIH selon la classification de l'OMS chez les enfants nés des mères VIH positives. L'infection urinaire était définie par la conjonction d'une leucocyturie et d'une bactériurie significatives. La méningite était définie sur base d'une pléiocytose dans le LCR > 5 éléments/mm^3^ associée au moins à une hyperprotéinorachie. En raison de l'indisponibilité des tests virologiques, la rougeole était définie par une fièvre associée à un catarrhe oculo-naso-pharyngé associée ou non au signe de Köplick avec un exanthème caractéristique.

L'abandon de traitement était défini par une absence au CNT pendant 3 jours consécutifs [8]. Le gain pondéral médian journalier a été calculé différemment selon qu'il s'agissait de la malnutrition sans œdème ou de la malnutrition avec œdème. Dans la malnutrition sans œdème, le gain pondéral médian journalier a été calculé par la formule:

poids (en gramme) à la sortie - poids (en gramme) au début de la rénutrition [9].

Poids (kilogramme) au début de la rénutrition x durée du traitement.

Dans la malnutrition avec œdème, il a été calculé par la formule:

poids (en gramme) à la sortie œ poids (en gramme) à la fonte des œdèmes [9].

Poids (en kilogramme) à la fonte des œdèmes x durée de la fonte des œdèmes.

Le gain pondéral médian journalier n'a pas été calculé chez les patients décédés et ceux ayant abandonné le traitement. Il a été uniquement calculé chez les survivants n'ayant pas abandonné le traitement. La classification utilisée était celle de l'OMS: gain pondéral médian journalier faible (< 5 g / kg / jour), gain pondéral médian journalier modéré (5-10 g / kg / jour) et gain pondéral médian journalier satisfaisant (> 10 g / kg / jour) [10]. La durée médiane de séjour a été calculée par la somme des jours (arrondis à 24 heures près) d'hospitalisation rapportée sur le nombre d'enfants de la population d’étude. Le volet descriptif de différentes variables est présenté sous forme des proportions, des pourcentages et des médianes avec espaces interquartiles (EIQ). Pour l'analyse statistique des données, nous avons utilisé le logiciel Epi-info 7.0.9.7. L’évaluation du risque de survenue de décès par une complication infectieuse a été faite par la mesure du risque relatif (RR). Pour la comparaison des médianes, nous avons utilisé le test de Mann-Whitney. Les Odd ratio (OR) ajusté par régression logistique et leurs intervalles de confiance à 95% du risque de mortalité ont été donnés pour chaque grande cause infectieuse. Ces différents tests ont été considérés comme statistiquement significatifs pour un p < 0.05.

## Résultats

Pendant la période d’étude, 859 enfants ont été admis au CNT, dont 294 enfants au cours de l'année 2011, 274 enfants en 2012 et 291 enfants en 2013. Cent trente d'entre eux présentaient la malnutrition aigüe modérée et faisaient partie de la fratrie des enfants admis pour MAS. Ils ont été exclus de l’étude. Cent cinquante-cinq enfants avec MAS avaient des dossiers incomplets, non exploitables. Ils ont également été exclus de l’étude. En définitive, seuls 574 enfants malnutris sévères ont été inclus. Leurs principales caractéristiques sont résumées dans le Tableau 1. Plus de la moitié (61.3%) de ces enfants étaient âgés de moins de 2 ans. L’âge médian était de 18 (12-30) mois. Trois cent cinq (53.1%) enfants étaient de sexe masculin et 269 (46.9%) étaient de sexe féminin, soit un sex-ratio de 1.1. La malnutrition avec œdèmes représentait 58% des cas. La durée médiane de séjour était de 19 (13-26) jours. Elle était longue chez les malnutris avec œdèmes et chez ceux avec plusieurs comorbidités associées, tel que le décrit le Tableau 2. Le gain pondéral médian journalier n'a pas été calculé chez les patients décédés (43 patients) et ceux ayant abandonné le traitement (10 patients). Chez les survivants qui n'ont pas abandonné le traitement (521 patients), le gain pondéral médian journalier était de 7 (3-13) g/kg/j. Comparativement aux enfants de 24 mois et plus, ceux de moins de 24 mois avaient un gain pondéral médian journalier élevé, tel que le résume le Tableau 3. Cinq cent vingt et un (90.8%) enfants étaient sortis guéris, 10 (1.7%) ont abandonné le traitement et 43 étaient décédés (7.5%). Le risque de décès était plus élevé chez les enfants malnutris infectés au VIH comparativement aux autres coïnfections. Après ajustement par régression logistique de facteurs de risque de la mortalité, seuls le sepsis, le choc septique, l'infection à VIH et la méningite gardaient une association statistiquement significative avec la mortalité comme le montrent les Tableau 4 etTableau 5.

**Tableau 1:** Association entre la mortalité et les variables démographiques, cliniques, biologiques et anthropométriques des enfants admis au *CNT pendant lap ériode d’étude

Variables	Survivants	décédés	Médiane (EIQ)	Total
**Age (mois)**	**531**	**43**		**574**
0 - 23	326	26	18 (12 – 30)	352
24 - 60	205	17		222
**Sexe**	**531**	**43**		**574**
Masculin	289	16		305
Féminin	242	27		269
**Œdèmes à l'admission**	**531**	**43**		**574**
Oui	311	22		333
Non	220	21		241
**Diarrhée à l'admission**	**531**	**43**		**574**
Oui	166	21		187
Non	365	22		387
**Hypothermie**	**531**	**43**		**574**
Oui	52	7		59
Non	479	36		515
**Rougeole**	**531**	**43**		**574**
Oui	19	2		21
Non	512	41		553
Sérologie **VIH	**531**	**43**		**574**
Positive	12	8		20
Négative	519	35		554
**Plusieurs comorbidités associées**	**531**	**43**		**574**
Oui	295	20		315
Non	236	23		259

* CNT : Centre Nutritionnel et Thérapeutique

** VIH : Virus de l'immunodéficience humaine

**Tableau 2:** Durées médianes de séjour des enfants admis au *CNT en fonction de leurs caractéristiques sociodémographiques, cliniques, biologiques et anthropométriques pendant la période d’étude

Variables	Médiane (EIQ) (jours)	n	p
	19 (13 - 26)		
Age (mois)		574	0,12
0 – 23	18 (12 - 26)	352	
24 – 60	20 (13 - 26)	222	
Sexe		574	0,69
Masculin	19 (13 - 26)	305	
Féminin	19 (13 - 26)	269	
Œdèmes		574	0,03
Oui	18 (11 - 26)	333	
Non	19 (14 - 26)	241	
Diarrhée		574	0,10
Oui	19 (13 - 26)	185	
Non	18 (11 - 25)	389	
Vaccin anti- rougeoleux		574	0,21
Oui	19 (13 - 26)	394	
Non	18 (11,5 - 26)	180	
Sérologie VIH*		574	0,07
Positive	13 (6,5 - 23,5)	20	
Négative	19 (13 - 26)	554	
comorbidités associées		574	0,03
Oui	18 (12 - 26)	316	
Non	20 (14 - 26)	258	

* CNT : Centre Nutritionnel et Thérapeutique

**VIH : Virus de l'Immunodéficience Humaine

**Tableau 3:** Gain pondéral journalier au cours du séjour des enfants malnutris en fonction de leurs caractéristiques sociodémographiques, cliniques, biologiques et anthropométriques pendant la période d’étude

Variables	Médiane (EIQ) (g/kg/j)	n	p
	7 (3 - 13)		
**Age (mois)**		**521**	**0,0001**
0 – 23	9 (5- 16)	319	
24 – 60	5 (3 - 10)	202	
**Sexe**		**521**	0,31
Masculin	8 (4 - 13)	319	
Féminin	7 (4 - 15)	202	
**Œdèmes**		**521**	0,32
Oui	10 (5 - 18)	306	
Non	6 (3 - 11)	215	
**Diarrhée**		**521**	0,55
Oui	9 (4 - 17)	165	
Non	7 (4 - 13)	356	
**Vaccin anti-rougeoleux**		**521**	0,66
Oui	7,5 (3 - 12)	394	
Non	7 (4 - 14)	180	
Sérologie VIH*		**521**	0,88
Positive	6 (4- 20,5)	18	
Négative	7 (4- 14)	503	
**Comorbidités associées**		**521**	0,06
Oui	8 (4,5 - 15)	277	
Non	7 (4 - 13)	244	

* VIH : Virus de l'immunodéficience humaine

**Tableau 4:** Association entre les complications infectieuses et la mortalité chez les enfants malnutris âgés de moins de 5 ans pris en charge au CNT*de l'HPGRB**pendant la période d’étude

Complications infectieuses		n total	n décès	% décès	RR (IC 95%)
Gastro-entérite	Présente	115	6	5,2	0,9 (0,9 - 1,1)
	Absente	442	37	8,4	
IVRS***	Présentes	25	3	12	1,0 (0,9 - 1,2)
	Absentes	533	40	7,5	
IVRI4*	Présentes	60	7	11,7	1,0 (0,9 - 1,1)
	Absentes	497	36	7,2	
Paludisme	Présente	82	5	6,1	0,9 (0,9 - 1,0)
	Absente	475	38	8	
Sepsis/ Choc septique	Présente	43	9	20,9	1,2 (1,1- 1,4)
	Absente	514	34	6,6	
VIH5*/Sida	Présente	20	8	40	1,6 (1,1- 2,2)
	Absente	546	35	6,4	
Rougeole	Présente	20	2	10	1,0 (0,9 - 1,2)
	Absente	546	41	7,5	
Infection urinaire	Présente	12	2	16,7	1,1 (0,8 - 1,4)
	Absente	554	41	7,4	
Tuberculose	Présente	8	0	0	0,9 (0,9 – 0,9)
	Absente	558	43	7,7	
Méningite	Présente	29	6	20,7	1,2 (1,0 -1,4)
	Absente	528	37	7	

* CNT : Centre Nutritionnel et Thérapeutique

** HPGRB : Hôpital Provincial Général de Référence de Bukavu

*** IVRS : infections des voies respiratoires supérieures

4* IVRI : infections des voies respiratoires inférieures

5* VIH : Virus de l'Immunodéficience Humaine

**Tableau 5:** Odd ratio ajusté par régression logistique defacteurs de risque de la mortalité chez les enfants malnutris âgés de moins de 5 ans pris en charge au *CNT de l'HPGRB**

Variables	OR ajusté IC (95%)	p-value
Gastro-entérite	0,6 (0,2 - 1,8)	0,42
IVRS***	2,2(0,6 - 8,2)	0,21
IVRI4*	1,3(0,4 - 3,4)	0,59
Paludisme	0,9 (0,3- 2,6)	0,90
Sepsis/ Choc septique	4,8(2,0 - 11,6)	**0,0004**
VIH5*/Sida	12,5 (4,4 - 35,4)	**<0,00001**
Rougeole	1,7 (0,3 - 8,3)	0,49
Infection urinaire	2,2(0,4 - 12,0)	0,34
Tuberculose	0,0(0,0 –1,12)	0,96
Méningite	30,4(1,6 - 573,7)	**0,02**

* CNT : Centre Nutritionnel et Thérapeutique

** HPGRB : Hôpital Provincial Général de Référence de Bukavu

*** IVRS : infections des voies respiratoires supérieures

4* IVRI : infections des voies respiratoires inférieures

5* VIH : Virus de l'Immunodéficience Humaine

## Discussion

### Biais de l’étude

Cent cinquante-cinq enfants avec MAS n'ont pas été retenus pour l’étude à cause de l'insuffisance d'informations dans les dossiers, ce qui représentait environ 20% de l'effectif assez important d'enfants éligibles mais non inclus. Il s'agit ici d'un biais de sélection qui pourrait avoir un impact sur l'extrapolation des conclusions de notre étude au cas où nos résultats s'avéraient complètement opposés aux données de la littérature.

### Aspects épidémiologiques

La classe d’âge de 0 à 23 mois était la plus affectée par la malnutrition et représentait plus de la moitié de l’échantillon. La prédominance de cette tranche d’âge est retrouvée par d'autres auteurs [11, 12]. En effet, c'est pendant cette période de la vie qu'interviennent la diversification alimentaire et le sevrage au lait maternel. La malnutrition survient quand cette diversification est inadéquate, faite précocement (avant 4 mois) ou tardivement (au-delà de 6 mois) ou avec des aliments de complément non adaptés, ou un sevrage total précoce. Pour une bonne croissance, un développement harmonieux et une santé optimale du nourrisson, l'OMS recommande l'allaitement maternel exclusif pendant les six premiers mois de la vie. Par la suite, pour répondre à l’évolution des besoins nutritionnels du nourrisson, ce dernier doit recevoir des aliments de complément sûrs et adéquats du point de vue de la composition nutritionnelle, tout en poursuivant l'allaitement jusqu’à l’âge de deux ans, voire plus [13]. Dans notre étude, il a été enregistré plus de garçons que de filles (sex-ratio = 1.1). D'autres études [14–16] avaient abouti aux mêmes résultats sans que la différence entre les deux sexes ne soit vraiment statistiquement significative.

### Aspects cliniques

Dans cette série, la malnutrition avec œdème était la forme prédominante de la MAS (58%). Cette observation corrobore les données anciennes de la Région du Kivu où la malnutrition avec œdème était plus répandue que le marasme [17]. Des études réalisées dans d'autres régions d'Afrique ont plutôt rapporté la prédominance de la MAS sans œdèmes, mais à des proportions assez variables, par exemple: 59.7% au Sénégal [11], 66.7% en Afrique du sud [18].

### Gain pondéral médian journalier et durée médiane de séjour

Le gain pondéral médian journalier des enfants hospitalisés au CNT est proche de ceux rapportés au Sénégal (7.6 g/kg/ jour) [11] et au Congo-Brazzaville (8.8 g/kg/jour) [12]. Il reste cependant de loin faible par rapport à celui obtenu dans une étude effectuée dans la sous-région de grands lacs africains en Tanzanie (15.0 g/kg/jour) [19]. Le gain pondéral médian journalier observé au CNT (7 g/kg/jour) est insuffisant selon Waterlow [20] qui a établi qu'un gain de 10 à 20 g/kg/jour était nécessaire pour une bonne réhabilitation nutritionnelle. L'OMS aborde dans le même sens en estimant qu'une réhabilitation nutritionnelle des enfants malnutris est satisfaisante si le gain pondéral quotidien est supérieur à 10 g/kg/jour [10]. Ce gain était plus élevé chez les enfants de moins de 24 mois comparativement aux enfants de 24 mois et plus. En effet, il est connu que chez les enfants de 0 à 24 mois, la vitesse de croissance est très rapide, alors qu'elle se ralentit entre 2 ans et le début de la puberté [21]. La durée médiane de séjour était de 19 jours et variait significativement en fonction des œdèmes et de la présence d'autres comorbidités associées. En effet, pendant la phase de réhabilitation nutritionnelle, les malnutris avec œdèmes connaissent d'abord une perte pondérale consécutivement à la fonte des œdèmes. L'ascension pondérale ne survient que par la suite. En revanche, les malnutris sans œdème gagnent directement le poids dès la phase de réhabilitation nutritionnelle. Ceci explique le long séjour dans la malnutrition avec œdème que dans celle où il n'y a pas d’œdème, car le gain pondéral est l'un des critères principaux de retour des enfants malnutris à leur domicile. Par ailleurs, les comorbidités contribuent grandement à l'allongement de la durée de séjour des malnutris.

### Pathologies infectieuses associées à la mortalité

Le taux de mortalité observé dans cette série était de 7.5%. Les pathologies infectieuses associées à un risque élevé de mortalité étaient entre autres le sepsis, le choc septique, la méningite et l'infection à VIH. Le bénéfice d'une antibiothérapie administrée précocement aux patients en état de sepsis sévère ou de choc septique a déjà été démontré. Kumar et al, dans leur étude visant à déterminer la prévalence et l'impact de l'initiation tardive des antibiotiques sur la mortalité dans le choc septique, ont rapporté une diminution de la mortalité chez les patients en état de choc septique lorsqu'une antibiothérapie adéquate leur était administrée dans l'heure suivant le début de l'hypotension artérielle. Ils ont même montré une augmentation croissante de la mortalité de 7.6% pour chaque heure du retard thérapeutique [22]. Par ailleurs, Puskarich et al [23] ont obtenu des résultats similaires, avec une diminution de la mortalité parmi les patients admis aux Urgences dans un tableau de choc septique, lorsque l'antibiothérapie était administrée dès l'apparition des signes de choc. Notre étude n'a pas évalué la corrélation entre la période d'initiation du traitement antibiotique et la mortalité dans le cadre de choc septique et de méningite. Cependant, la mortalité liée au choc septique et à la méningite pourrait être en partie attribuable à la gravité intrinsèque de ces deux entités nosologiques. Enfin, l'infection à VIH aggrave davantage l'immunodépression déjà présente dans la malnutrition. Elle accroît la prévalence de la malnutrition aiguë sévère [24, 25]. En effet, une étude a montré que plus de 30% des enfants sévèrement malnutris en Afrique subsaharienne hospitalisés dans des unités de réhabilitation nutritionnelle étaient infectés par le VIH [26]. D'autres études [27–31] ont montré que la mortalité était plus importante chez les enfants malnutris infectés au VIH. Ces derniers souffraient entre autres de diarrhée persistante, de pneumonie, d'infections dermatologiques étendues et de candidose buccale; et toutes ces affections contribuaient à un taux de mortalité plus élevé et à une évolution moins favorable à la prise en charge [27–31].

## Conclusion

Il est incontestable que les maladies infectieuses aggravent la malnutrition en contribuant à l'affaiblissement du système immunitaire de l'enfant. Dans cette série des cas de MAS, le sepsis, le choc septique, la méningite et l'infection à VIH se révèlent comme étant les infections les plus meurtrières chez les enfants malnutris. Ainsi, une meilleure prise en charge de la MAS dans notre région devrait se baser sur la mise en place des unités spécialisées et bien équipées pour la prise en charge des cas les plus graves de MAS, en l'occurrence celles compliquées ou associées à certaines infections sévères ou chroniques.

### Etat des connaissance sur le sujet

- La malnutrition est immunodépressive; en cas de malnutrition, les infections sont plus fréquentes, plus prolongées et plus graves.
- Plus de 70% des décès d'enfants< 5 ans résultent de l'association malnutrition-infection.

### Contribution de notre étude a la connaissance

- L’étude détermine les types d'infections associées de façon statistiquement significative à la mortalité des enfants malnutris: sepsis, choc septique, méningite, infection à VIH.
- L’étude compare le gain pondéral médian journalier des enfants malnutris infectés par rapport aux normes de l'OMS: ce gain est faible.
